# Code Blue: Life-threatening Methemoglobinemia

**DOI:** 10.5811/cpcem.2019.3.41794

**Published:** 2019-03-27

**Authors:** José D. Ponce Ríos, Rothsovann Yong, Paul Calner

**Affiliations:** * Lowell General Hospital, Department of Pediatric Emergency Medicine, Lowell, Massachusetts; † Lowell General Hospital, Department of Emergency Medicine, Lowell, Massachusetts

## Abstract

Most cases of acquired methemoglobinemia result from exposure to certain drugs or toxins. One of the more common and well-described causes in the literature is exposure to topical benzocaine during medical procedures. We present a case series of acute acquired methemoglobinemia from a food source that has not been previously described in the literature: a dessert. Three patients, ages 5, 33, and 86 years, were brought to our emergency department by ambulance after becoming extremely ill from ingesting a dessert containing nitre powder at a family gathering. They all presented with hypotension, cyanosis, and hypoxia that was not responsive to oxygen administration. The adult patients had major improvement of symptoms after a single dose of methylene blue. In contrast, the 5-year-old child who had the worst symptoms minimally improved with administration of two doses of methylene blue requiring intensive care admission and transfer to a tertiary care center.

## INTRODUCTION

Methemoglobinemia, which can be congenital or acquired, occurs when there is an elevated level of methemoglobin circulating in the blood. Methemoglobin is an oxidized form of hemoglobin;^1^ it is produced when the iron in the hemoglobin molecule is in the ferric instead of the ferrous state. Methemoglobin, unlike its counterpart oxyhemoglobin, cannot carry oxygen or carbon dioxide and therefore leads to a state of hypoxia. Clinical manifestations include altered mental status, cyanosis, acidosis, impaired aerobic respiration, and in severe cases, coma and death.^2^ We report our experience with three patients who presented to the emergency department (ED) with acquired methemoglobinemia after ingestion of a dessert at a family gathering. We will discuss diagnosis and management for methemoglobin toxicity.

## CASE SERIES

Three patients, ages 5, 33 and 86 years, presented to the ED after becoming acutely ill at a family gathering. All three members ingested a pandan honeycomb cake, which contained “nitre powder” used as a thickening agent. While all three patients had significant symptoms, here we present in detail the case of the 5-year-old child who had the most profound symptoms.

## CASE 1

**Image 1:**
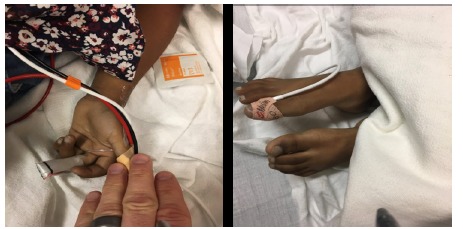
Five-year-old patient’s severely cyanotic hand and feet.

A 5-year-old female who fell ill after eating dessert at a party presented to our ED via ambulance. The parents reported that the child had two large servings of cake and subsequently developed vomiting, loose stools, and syncope. They noted her to be “blue” (Image 1); 911 was called, and she was brought by ambulance to the hospital. During our encounter, the patient was very quiet and did not report any complaints. Vital signs upon arrival to the ED showed blood pressure 75/47 millimeters of mercury (mmHg), pulse oximetry 77% on 12 liters non-rebreather mask, respiratory rate 30 breaths per minute, and heart rate of 117 beats per minute. Her physical exam was significant for cyanosis, clear lungs to auscultation, and tachycardia on cardiovascular examination. The diagnosis of methemoglobinemia was quickly suspected given the clinical presentation of sudden onset cyanosis not improving with oxygen administration as well as low pulse oximetry.

Methemoglobinemia workup was initiated; upon collection, blood appeared very dark in the laboratory tubes. Co-oximetry confirmed an elevated methemoglobin level at 52%, venous blood gas showed a partial pressure of oxygen (pO_2_) of 178 mmHg, and lactic acid was 3.3 millimoles per liter (mmol/L). Blood pressure improved after two 20 milliliters per kilogram (mL/kg) boluses of normal saline, but patient continued to have tachycardia and cyanosis. We administered one milligram per kilogram (mg/kg) of methylene blue intravenously over five minutes. When we re-checked her methemoglobin level at 30 minutes after methylene infusion, it was 38.6%; pulse oximetry remained in the 70%-80% range. The patient continued to appear cyanotic despite administration of the first dose of methylene blue; therefore, we administered the second dose of one mg/kg of methylene blue. While the patient had some improvement, she continued to be cyanotic throughout her course in the ED.

Due to a persistently elevated methemoglobin level, continued cyanosis, tachycardia, and signs of hypoxia (Table), despite the second dose of methylene blue, the decision was made to admit the child to a tertiary care center. She was transferred to the facility 60 minutes away by the tertiary care center’s critical care ground transport team, which included a pediatric intensive care nurse and pediatric respiratory therapist, as well as two paramedics. Upon arrival, her methemoglobin level was 6.7% and lactate level was normal; supplemental oxygen was removed and remained off overnight. She tolerated oral intake and was discharged home the next day. No additional methemoglobin level was obtained.

## CASE 2

A 33-year-old female with no significant past medical history presented to our ED from the same party after ingesting the same dessert. She reported feeling short of breath and had vomiting, loose stool, and a near-syncope event. She had no chronic conditions, took no medications on a daily basis, and had no allergies. Upon arrival, she appeared in mild distress and slightly anxious. Vital signs in the ED showed blood pressure 99/75 mmHg, pulse oximetry 81% on six liters nasal cannula, a respiratory rate of 16 breaths per minute, and heart rate of 87 beats per minute. Except for cyanosis on physical exam, the patient appeared in no acute distress. She had clear lungs, normal cardiovascular exam and an unremarkable abdominal exam. She had a methemoglobin level of 17.2%. After she was treated with one mg/kg methylene blue, vital signs normalized, and repeat methemoglobin level three hours after methylene blue infusion was 1.2%. She was discharged from the hospital after several hours of observation.

## CASE 3

An 86-year-old female with a past medical history of hypertension and dyslipidemia was brought to our ED by ambulance from the same family gathering after ingesting the same honeycomb cake. She complained of feeling unwell with lightheadedness, headache, recurrent vomiting, shortness of breath, and chest pain followed by a syncopal episode. Paramedics reported that the patient was cyanotic with pulse oximetry 70% on 12 liters non-rebreather mask and hypotensive with systolic blood pressure 80 mmHg. On arrival to the ED, she was awake, alert and ill-appearing with the following vital signs: pulse oximetry 85% on non-rebreather mask, respiratory rate 25 breaths per minutes, pulse rate 98 beats per minutes, and blood pressure 115/72 mmHg after one liter normal saline bolus. Her physical exam was significant for severe cyanosis, tachypnea, clear lungs to auscultation, and tachycardia on cardiovascular examination. Given the concurrent presentation with the other two patients from the same party, we treated the patient with one dose of methylene blue prior to obtaining initial methemoglobin levels.

**CPC-EM Capsule**

What do we already know about this clinical entity?

*In methemoglobinemia, high levels of methemoglobin circulate in the blood; if untreated, it can lead to death. Symptoms include cyanosis, tachycardia, shortness of breath, and lethargy*.

What makes this presentation of disease reportable?

*We represent a case series of acute acquired methemoglobinemia from a food source that has not been previously described in the literature: a dessert*.

What is the major learning point?

*Methemoglobinemia can be fatal. Prompt administration of methylene blue should be the first line treatment; ascorbic acid can be used as an adjuvant*.

How might this improve emergency medicine practice?

*Methemoglobinemia diagnosis requires high index of suspicion thorough history and physical exam. Prompt identification of patients is paramount to minimize morbidity and mortality*.

Her vital signs stabilized on reevaluation after 30 minutes of methylene blue administration. She reported complete resolution of symptoms including chest pain, shortness of breath, and headache. Her pulse oximetry improved to 92% on room air. Her comprehensive metabolic panel results were within normal limits except for a slight elevation of creatinine from her baseline. Troponin I levels were negative. Initial methemoglobin levels pretreatment were unknown, but levels obtained at 30 minutes and 10 hours after methylene blue administration were 6.7% and 0.7%, respectively. The patient was admitted to telemetry for further observation, but once there she became hypotensive with blood pressure 90/50 mmHg despite administration of two additional liters of normal saline. She was then transferred to the intensive care unit (ICU) for monitoring. She remained asymptomatic on room air with resolution of hypotension after receiving intravenous fluids at 100 mL per hour in the ICU overnight and was downgraded back to telemetry in the morning. She had a full recovery and was discharged home the following day.

## DISCUSSION

Methemoglobinemia can be congenital or acquired. There are four types of congenital methemoglobinemia.^3^ Type I (the most common) is cytochrome b5 reductase deficiency in the red blood cells, leading to mild cyanosis, headaches, and fatigue. In type II there is a global deficiency of cytochrome b5 reductase, and patients have severe symptoms as well as neurologic deficits.^3^–^5^ Type III is no longer considered an entity of its own as it presents like type I; type IV has only been described once.^4^,^6^,^7^

Acquired methemoglobinemia can occur from a myriad of causes; medications and oxidation stress by illness have all been described in the literature. Some xenobiotics are commonly identified as culprits in cases of acquired methemoglobin. These agents include dapsone, lidocaine, nitrates, and sulfa-containing drugs.^8^–^11^

Acquired methemoglobinemia presents with multiple symptoms secondary to a lack of oxygen delivery to tissues. These symptoms include cyanosis, tachycardia, shortness of breath and lethargy as we saw in our cases. As the patient’s methemoglobin level increases, he or she will develop changes in mental status and increased respiratory rate, and potentially coma, seizures, and even death.^12^,^13^

It is typically only after the methemoglobin level exceeds 10% of total hemoglobin that patients will become cyanotic. Levels greater than 30–40% are considered life-threatening, similar to what we found in our 5-year-old patient, and are associated with severe symptoms and profound hypoxia. Levels greater than 70% are often fatal.^1^,^13^–^16^

Methemoglobinemia should be suspected in all patients with sudden onset of cyanosis that does not improve with the administration of oxygen or after ingestion or administration of a potential oxidative agent. It should also be suspected in patients who are clinically cyanotic but have a normal arterial pO_2_. An additional clue in our 5-year-old patient that helped us diagnose methemoglobin toxicity was discoloration of the blood sample observed during phlebotomy. Blood with an elevated methemoglobin level has been described as chocolate, brownish-blue, or dark red in color.^12^ Once suspected, a methemoglobin level should be obtained. It is important not to follow pulse oximetry on these patients as the value is often inaccurate.^2^

Once identified, methemoglobinemia should be treated aggressively, especially if methemoglobin levels are greater than 20% or if the patient is symptomatic.^1^ There are two main treatments available: methylene blue and ascorbic acid.^17^ In patients with acute acquired methemoglobinemia who are symptomatic or have methemoglobin levels > 20 a single dose of one to two mg/kg of methylene blue should be given over five minutes, within 10–60 minutes of symptom onset. The response is typically very quick and a second dose is rarely required except in severe cases.^18^ In severe methemoglobinemia, patients should be managed in an ICU setting for close monitoring and stabilization of their airway, breathing, and circulation. If a patient has a methemoglobin level < 20 % and is asymptomatic, no therapy is recommended and close observation is reasonable. Alternatively, ascorbic acid may be used to treat acquired methemoglobinemia, especially in those patients with glucose-6-phosphate dehydrogenase deficiency (G6PD) where methylene blue is contraindicated, or, if methylene blue is not available. Therapeutic doses of methylene blue may lead to severe hemolysis of erythrocytes especially in patients with G6PD; if ascorbic acid is used, multiple doses are often required over 24 hours or more.^17^,^19^,^20^

**Image 2:**
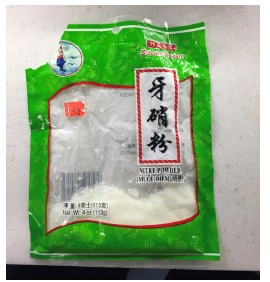
Package of nitre powder purchased from a local supermarket.

Upon further investigation with the family we discovered that the dessert had “nitre powder,” which had been used as a thickening agent. “Nitre powder” is actually sodium nitrate (Image 2), a crystalline powder used for curing meats and preserving their color. The United States Food and Drug Administration allows its use for curing meats.^21^ In our literature review of acquired methemoglobinemia, we found cases of nitrate ingestions from ingesting cured meats at home^22^ and in intentional ingestions,^15^ but no cases related to ingesting a dessert. Obtaining a thorough medical history and physical exam was paramount in obtaining a diagnosis for these three patients. These cases were reported to the Department of Public Health since the product had been recently purchased at a local market despite it having been recalled years prior due to safety concerns.

## CONCLUSION

Methemoglobinemia should be suspected in all patients with sudden onset of cyanosis that does not improve with the administration of oxygen. Prompt administration of methylene blue should be the first-line treatment. Ascorbic acid can be used as adjuvant therapy when there is a contraindication to methylene blue, or when methylene blue is not available.

## Figures and Tables

**Table:** Laboratory data and vital signs pre- and post-treatment with methylene blue.

Venous blood gas (100% FiO_2_)	Pretreatment	30 Minutes post treatment	Reference range
pCO_2_ (mmHg)	34.4	39.2	41.0–51.0
pO_2_(mmHg)	178.0	41.4	
HCO_3_(mmol/L)	*21.9	*19.4	22.0–26.0
pH	7.382	*7.317	7.320–7.420
Carboxyhemoglobin (%)	0.1	0.4	
Methemoglobin (%)	*52.0	*38.6	< 2
Lactic acid (mmol/L)	*3.3	Not obtained	0.4–2.0
Red blood cells 10^9^	3.82	Not obtained	3.7–5.30
Hemoglobin (g/dL)	10.9	Not obtained	11.0–14.0
Hematocrit(%)	32.8	Not obtained	33.0–42.0
Pulse rate (beats per minute)	117	*129	90–120
Respiratory rate (breaths per minute)	*30	26	20–28
Systolic blood pressure (mmHg)	*75	91	89–112
Diastolic blood pressure (mmHg)	47	*40	46–72
SpO_2_ on 100% supplemental oxygen (%)	*77	*82	> 90

pCO_2_, carbon dioxide partial pressure; pO_2_, oxygen partial pressure; HCO_3_, bicarbonate; mmHg, millimeters of mercury; mmol/L, millimoles per liter; g/dL, grams per deciliter; SpO_2_, saturation of peripheral oxygen; FiO_2_, fraction of inspired oxygen.

* Abnormal findings.
